# Intranasal Drug Administration for Psychomotor Agitation as a Safe and Effective Prehospital Intervention: An Integrative Review

**DOI:** 10.3390/nursrep15060219

**Published:** 2025-06-16

**Authors:** Amaya Burgos-Esteban, Valvanera Cordón-Hurtado, Marta Giménez-Luzuriaga, Maria Peinado-Quesada, Laura Gómez-Lage, Raúl Juárez-Vela, Michal Czapla, Jorge García-Criado, Noelia Navas-Echazarreta, Antonio Rodríguez-Calvo, Pablo Lasa-Berasain, Manuel Quintana-Diaz

**Affiliations:** 1 Doctoral Program in Medicine and Surgery, Faculty of Medicine, Autonomous University of Madrid, 28049 Madrid, Spain; amaya.burgos@unirioja.es; 2 Hospital San Pedro, 26006 Logroño, Spain; macordh@gmail.com; 3 Department of Nursing, University of La Rioja, 26006 Logroño, Spain; marta.gimenez@unirioja.es (M.G.-L.); noelia.navas@unirioja.es (N.N.-E.); 4 Intensive Care Unit, Hospital La Paz, 28046 Madrid, Spain; mariaangustias.peinado@salud.madrid.org (M.P.-Q.); laura.gomez2@salud.madrid.org (L.G.-L.); manuel.quintana@salud.madrid.org (M.Q.-D.); 5 IdiPaz Research Institute, PBM Group, Hospital La Paz, 28046 Madrid, Spain; 6 Department of Emergency Medical Service, Faculty of Nursing and Midwifery, Wroclaw Medical University, 51-618 Wroclaw, Poland; 7 Complex Hospital University of Salamanca, Faculty of Medicine, University of Salamanca, 37008 Salamanca, Spain; jgarciacr@saludcastillayleon.es; 8 Department of Anesthesiology, University Hospital of Salamanca, 37007 Salamanca, Spain; arodriguezc@saludcastillayleon.es; 9 Intensive Care Unit, Hospital of Navarra, 31008 Pamplona, Spain; pablo.lasa.berasain@navarra.es

**Keywords:** intranasal drug administration, psychomotor agitation, out-of-hospital settings, emergency medical services, emergency nursing

## Abstract

**Introduction:** Psychomotor agitation represents a complex medical emergency, particularly challenging in prehospital settings. Since March 2020, the incidence of psychomotor agitation has significantly increased. **Rationale:** Emergency Medical Services (EMS) frequently serve as the first point of contact, bearing the critical responsibility of effectively managing these situations. **Objective:** This was to assess the feasibility and suitability of the intranasal route for administering pharmacological therapy in the prehospital management of patients experiencing psychomotor agitation. **Materials and Methods:** An integrative review of the literature was conducted to evaluate the use of the intranasal route for drug administration in patients with psychomotor agitation in prehospital settings. The review was carried out between September 2022 and July 2024. A total of 454 articles were identified, 15 of which met the inclusion criteria. These were supplemented by an additional 10 records, resulting in the analysis of 25 studies. **Results:** Seventeen studies outlined protocols for managing agitated patients, five described the correct technique for intranasal drug administration, and eleven identified drugs suitable for this route. **Conclusions:** The intranasal route is a safe, rapid, and accessible method for the pharmacological containment of agitated patients in prehospital settings, particularly for individuals who are uncooperative.

## 1. Introduction

Mental health is a fundamental right recognized by the World Health Organization (WHO) and is essential for the well-being and development of individuals and communities [1]. However, the WHO highlights that many healthcare systems fail to allocate sufficient attention and resources to mental health [1]. In Spain, the National Health System (NHS) ensures comprehensive and continuous mental healthcare through its standardized portfolio of services [2]. Globally, one in eight individuals suffers from some form of mental health disorder, with depressive and anxiety disorders being the most prevalent. Suicide accounts for 1 in every 100 deaths worldwide, while mental disorders are the leading cause of years lost due to disability (YLD), contributing to one in every six cases [1]. Psychomotor agitation is defined as a state in which patients exhibit disorganized and excessive psychomotor activity [3]. This condition can have organic, psychiatric, or mixed origins [3,4,5]. During episodes of psychomotor agitation, individuals may display behaviors characterized by anxiety, euphoria, or agitation, often accompanied by uncontrolled laughter, crying, or shouting. These behaviors can escalate into verbal and/or physical aggression, posing risks to the individual (e.g., self-harm), as well as to others, including family members, healthcare providers, and the surrounding environment [6]. Psychomotor agitation constitutes a medical and psychiatric emergency that is frequently managed in out-of-hospital settings by Emergency Medical Services (EMS) [4,7,8,9]. Immediate intervention is essential to control the patient’s symptoms and mitigate risks to the patient, healthcare personnel, and the environment [4,10,11].

The underlying cause of these symptoms is often associated with a mental health disorder [3]. In Spain, 3% of the population suffers from a severe mental health disorder, 9% experience some form of mental health issue, and 25% will face such a condition at some point in their lives [12].

The COVID-19 pandemic has exacerbated what has been termed a “global crisis” in mental health [1]. Anxiety and depressive disorders increased by more than 25% during the first year of the pandemic, further widening the gap in the therapeutic management of mental health disorders and associated episodes of psychomotor agitation.

The management of an agitated patient should prioritize controlling behavior, conducting a comprehensive medical evaluation, and determining the most appropriate treatment [8]. Non-cooperation from patients can complicate emergency interventions, necessitating early therapeutic measures guided by a structured system and individualized patient assessment [7]. General measures for the out-of-hospital management of psychomotor agitation include the implementation and maintenance of safety protocols, verbal de-escalation, mechanical restraint, and pharmacological intervention [3,4,5,13]. In some cases, the patient’s lack of cooperation increases the risks associated with forced drug administration. The intranasal route for pharmacological therapy offers healthcare professionals a rapid, straightforward method of administration while providing a less invasive and more tolerable option for patients [4,9,14].

The aim of this study is to review the current data on the intranasal route for administering pharmacological therapy in the out-of-hospital management of patients with psychomotor agitation.

## 2. Materials and Methods

### 2.1. Methodology

The route of administration for pharmacological therapy in patients presenting with psychomotor agitation in out-of-hospital settings was analyzed from September 2022 to July 2024 using the search engines and databases PubMed, Dialnet, ENFISPO, and Google Scholar. Inclusion Criteria: The analysis included cross-sectional descriptive scientific articles, case series, randomized clinical trials, systematic reviews, literature reviews, protocols, clinical guidelines, and action manuals that addressed, in general, the management of agitated patients and/or the use of the intranasal route for pharmacological therapy. Exclusion Criteria: Articles classified as editorials, clinical notes, or letters to the editor were excluded, as well as studies focusing on the use of the intranasal route for clinical conditions other than psychomotor agitation.

### 2.2. Search Strategy

The initial search was conducted by a single researcher, while the selection of articles was performed independently by two researchers. Subsequently, each reviewer reevaluated the selected articles for inclusion, and any discrepancies were resolved by a third reviewer. Additionally, Spanish legislation and the 2022 World Health Organization Mental Health Report were analyzed to contextualize the current situation. The search terms included both free language and controlled vocabulary. In free language, terms such as “agitated patient,” “intranasal route,” “out-of-hospital emergency medical services,” “alternative method,” and “out-of-hospital psychiatric emergencies” were used. For controlled vocabulary, Medical Subject Headings (MeSH) terms included “psychomotor agitation,” “drug administration, intranasal,” “emergency medical services,” and “drug therapy.” Similarly, DeCS terms included “psychomotor agitation,” “intranasal administration,” and “drug therapy.” Logical relationships between these terms were established using the Boolean operator “AND.”.

### 2.3. Search Strategy Design

The search strategy (Table 1) was developed based on the research question formulated in PICO format: Can the use of the intranasal route for the administration of drug therapy improve the quality of care and safety in the out-of-hospital management of patients with psychomotor agitation? (Table 2). This integrative approach ensured a comprehensive and rigorous review of the available evidence, aligning with the study’s objective to evaluate the suitability of the intranasal route for pharmacological therapy in out-of-hospital settings.

### 2.4. Data Collection

The data were collected using a pre-designed template that included the following key information: the author, year, type of study, and scope of application for therapeutic measures; indications for pharmacological containment in the management of agitated patients; devices for intranasal drug administration; recommendations for the use of the intranasal route; drugs indicated for the containment of agitated patients; and research variables.

The study focused on the following research variables:

Steps to Follow in the Management of the Agitated Patient: These included safety protocols, verbal de-escalation, pharmacological restraint, and mechanical restraint. Technique for the Administration of Intranasal Therapy: This included precautions, contraindications, administration devices, recommended volume, maximum volume, time to onset of action, and time to peak action. Drugs Indicated for the Pharmacological Restraint of Psychomotor Agitation Suitable for Intranasal Administration: These included benzodiazepines, neuroleptics, and other pharmacological agents.

#### 2.4.1. Identification of Articles

The search process initially retrieved a total of 627 articles from the following databases:

- PubMed, 199 articles;
- Dialnet, 100 articles;
- ENFISPO, 35 articles;
- Google Scholar, 293 articles.

After eliminating duplicate articles [10] and excluding those that did not meet the established inclusion and exclusion criteria (579), the titles and abstracts of the remaining 38 articles were reviewed. Based on their relevance to the study criteria, 15 studies were selected for detailed reading and subsequently incorporated into the investigation.

In addition to these studies, 10 official reports retrieved from recognized web pages were also included to provide additional context and information.

#### 2.4.2. Final Selection

**Figure 1:**
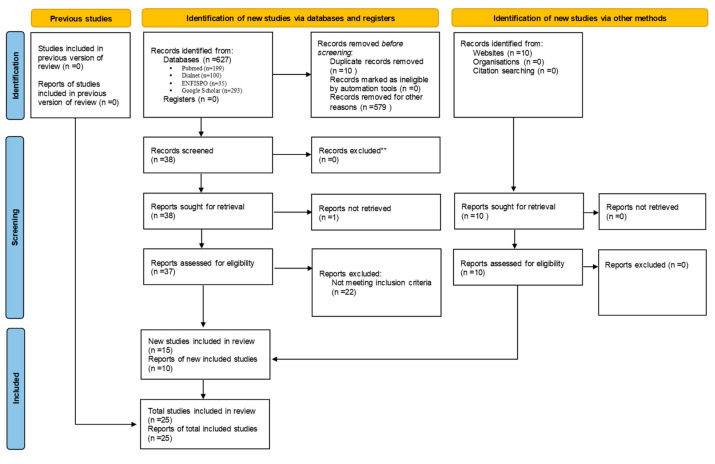
Flow chart. **: no records excluded, just for highlighted it.

Following this rigorous process of identification, selection, and inclusion, a total of 25 texts were ultimately included in the study. This process is visually summarized in the attached flow chart (Figure 1).

This integrative approach ensured the inclusion of high-quality, relevant evidence to address the study’s objectives.

## 3. Results

A total of twenty-five studies published between 2012 and 2024 were included in this analysis: one clinical practice guideline [11], one action guideline [15], five procedure manuals [5,16,17,18,19], seven action protocols [20,21,22,23,24,25,26], two clinical trials [27,28], three observational studies (two retrospective [29,30] and one prospective [31]), four literature reviews [32,33,34,35], one systematic review [36], and one consensus document [37].

The content analysis (Table 3) revealed the following:

Steps for managing psychomotor agitation: we found that 17 of the 24 texts analyzed specify the steps to be followed [5,15,16,17,19,20,21,22,23,24,25,26,29,33,34,36,37].

Correct technique for intranasal drug administration: Five texts address this topic [11,16,18,31,32].

The intranasal administration of drugs for the pharmacological management of agitation: Eleven texts discuss the possibility of using this route [5,11,18,20,27,28,29,30,32,34,35].

Scope of Care (Table 4): Of the 17 articles reviewed on the steps to manage an agitated patient, 8 refer to care in the prehospital setting [5,17,20,21,24,25,27,30]. Five refer to care at the hospital level [16,18,23,26,37]. Four describe patient care without specifying the area of intervention [22,34,35,38].

Protocols Analyzed: The study included an analysis of the protocols currently in force in the Autonomous Communities of Andalusia, Aragon, Asturias, Castile and Leon, Galicia, La Rioja, and Madrid, including the Servicio de Urgencias Médicas de Madrid (SUMMA) and the Servicio de Asistencia Municipal de Urgencia y Rescate (SAMUR) [5,17,20,21,24,25,26,27]. The combined population of these communities totals 23,070,528 inhabitants [38].

### 3.1. Steps to Follow in a Patient Presenting with Psychomotor Agitation

An exhaustive analysis of the selected texts highlights several key findings, which will now be presented.

#### 3.1.1. Determining the Origin of the Condition

Twelve sources emphasize the importance of identifying whether the condition is psychiatric, organic, or mixed in nature [5,16,17,18,20,22,24,25,27,35,37,38]. The Galician Health Service protocol for out-of-hospital emergency intervention underscores the need for a brief history, physical examination, psychiatric evaluation, and identification of vital risk signs [26].

#### 3.1.2. Ensuring Safety

Thirteen studies prioritize measures to ensure the safety of the patient, their family, and the healthcare team [5,16,17,20,21,23,24,25,27,34,35,37,38]. These measures include risk evaluation [5] and creating a safe, trust-promoting environment [21]. Recognizing prodromal symptoms is highlighted as a preventive step for avoiding escalation [16,18,34], allowing for the control of mild and moderate symptoms, as noted by the Spanish Society of Psychiatry [38]. The SAMUR protocol also stresses minimizing unnecessary stimuli [17].

#### 3.1.3. Verbal De-Escalation

Fourteen studies identify verbal de-escalation as a critical tool for managing agitation [5,16,18,20,21,22,23,24,25,26,34,35,37,38]. Verbal strategies are effective in promoting de-escalation [22]. The ANESM and the SEEUE, in their 2016 consensus document, highlight the importance of active listening and avoiding confrontation to redirect the situation [35]. The Spanish Society of Psychiatry also emphasizes the use of verbal and non-verbal communication techniques [38].

#### 3.1.4. Pharmacological Management

All 17 analyzed articles support pharmacological treatment as a cornerstone in managing psychomotor agitation [5,16,17,18,20,21,22,23,24,25,26,27,30,34,35,37,38]. Benzodiazepines [5,16,17,18,21,23,24,25,26,27,35,38], neuroleptics [6,16,17,18,21,23,24,25,26,27,35,38], or a combination of both [18,23,24,26,27,35] are commonly recommended for their rapid onset and reduced adverse effects [18,35].

The EMS protocol of La Rioja mentions propofol as an additional sedative–hypnotic option [21].

Huebinger et al., in a retrospective observational study, support the use of midazolam and ketamine for pharmacological containment [30].

The Spanish Society of Psychiatry advises against combining multiple drugs and recommends voluntary administration via oral, sublingual, or intranasal routes, minimizing parenteral administration to maintain the therapeutic relationship [38].

The ANESM suggests that the route of administration—oral, inhalation, or parenteral—should depend on the patient’s level of cooperation [35].

E. Martínez Larrull et al. advocate for the oral route as the preferred option [23].

Guidelines from the Spanish Society of Psychiatry recommend short-acting drugs at minimal effective doses [38].

The Galician Health Service protocol emphasizes tailoring treatment to the patient’s level of agitation [25], with the ultimate goal being to help the patient achieve a calm state [37].

#### 3.1.5. Physical or Mechanical Restraint

Sixteen of the seventeen texts recognize physical or mechanical restraint as a potential step in managing severe agitation or uncontrollable impulsivity, but only in exceptional cases [5,16,17,18,20,21,22,23,24,25,26,27,34,35,36,37,38]. The primary objective is to ensure the safety of the patient, the environment, and healthcare professionals [21,26].

The 061 Emergency Protocol of La Rioja highlights the importance of staff training and teamwork for effective intervention [21], a recommendation echoed by P. Sanz Correcher [22].

The ANESM and SEEUE advocate for the use of approved restraint devices [35].

J.I. Gallego-Gómez et al. identify situations where mechanical restraint may be necessary as the first therapeutic measure [37].

The SUMMA protocol notes that mechanical restraint is often combined with pharmacological restraint [5].

The Coordination Protocol for the Care of Agitated Patients from the Government of Aragon stresses the importance of an immediate response to agitation-related symptoms [24].

See Table 5 for further details.

### 3.2. Key Points for Intranasal Drug Delivery: Recommendation of the Atomizer Devices

An analysis of five articles addressing the proper technique for intranasal drug administration highlights the following recommendations:

#### 3.2.1. Use of Atomizing Devices

All five articles recommend using an atomizing device for intranasal drug delivery [11,17,19,32,33]. Three of these emphasize that the device improves both drug distribution and absorption [11,19,33]. The Spanish Society of Pediatrics (SEUP) notes that this conclusion is based on expert opinion [19]. The Emergency Nurses Association (ENA) highlights that the intranasal route is suitable for both children and adults, in hospital and out-of-hospital settings [11]. This route is particularly indicated when venous access is difficult [11,19] or oral administration is not feasible [11].

#### 3.2.2. Factors Affecting Absorption

Several factors can interfere with drug absorption via the intranasal route:

The presence of mucus or blood, as well as nasal septum anomalies, can reduce absorption [11,17].

The ENA states that excessive mucus or blood contraindicates this route [11].

SAMUR notes that the prior use of vasoconstrictors can negatively impact absorption [17].

The SEUP lists contraindications, including epistaxis, recent nasal trauma, septal abnormalities, impaired ciliary function, mucus, hematomas, nasal polyps obstructing the nasal cavity, the prior use of vasoconstrictors, or allergy to the drug [19].

#### 3.2.3. Correct Technique for Atomizer Use

Volume Per Nostril: All five texts agree that the ideal volume per nostril is between 0.2 and 0.3 mL [11,17,19,32,33]. However, the ENA, Tucker C et al., and SAMUR state that up to 1 mL can be administered if necessary [11,17,33]. For larger volumes, administration should be repeated, with a 5 to 15 min interval between doses [19,33].

Dead Space Compensation: When preparing the first dose, an additional 0.1 mL should be added to account for the atomizer’s dead space [19,32,33].

Dose Division: The total dose should be divided into two syringes [19,32,33], with half administered per nostril [17,19,32,33].

#### 3.2.4. Procedure Steps

Inform the patient about the procedure beforehand [19,32].

Check and clean the nostrils before atomization [19,32].

Insert the atomizer into the nostril and propel the contents over one to two seconds [32], keeping the cone in place for approximately 5 to 10 s [19].

The onset of action occurs within two to three minutes, with maximum effect achieved in 10 to 15 min [33].

#### 3.2.5. Precautions and Protocols

The SEUP protocol emphasizes that the same precautions as for other drug administration routes should be followed. These include verifying the absence of allergies or contraindications, preparing the necessary materials, ensuring the correct patient, drug, route, dose, and administration time, and informing the patient, family, and physician about the procedure [19]. See Table 6 for a summary of the key points.

### 3.3. Pharmacological Indications

An analysis of ten articles reveals the following key points regarding the intranasal administration of drugs for managing psychomotor agitation:

#### 3.3.1. Intranasal Midazolam

Eight of the ten articles analyzed recognize the appropriateness of intranasal midazolam for managing agitation [11,19,21,28,29,30,31,33,35,36].

The National Association of Mental Health Nursing (ANESM) and the Spanish Society of Emergency Nursing (SEEUE) recommend midazolam for both intranasal and intravenous administration [35].

Huebinger RM et al. suggest intramuscular administration as an alternative [30].

A review article published in 2023 by the Chilean Journal of Anaesthesia concludes that intranasal midazolam achieves plasma levels comparable to intravenous administration, with an appropriate dose of 0.2 mg/kg.

#### 3.3.2. Intranasal Ketamine

The same Chilean review highlights the use of intranasal ketamine for psychomotor agitation, noting an onset of action between 5 and 10 min [36].

Duñó Ambros R et al., in a randomized clinical study, recommend intranasal ketamine for out-of-hospital settings [28].

#### 3.3.3. Intranasal Haloperidol

Duñó Ambros R et al. emphasize the suitability of intranasal haloperidol for managing mild-to-moderate agitation in hospital settings [28].

Tucker C et al. note that parenteral formulations achieve good results when used intranasally but do not delve into haloperidol due to limited evidence supporting its clinical use [33].

#### 3.3.4. Intranasal Olanzapine

Shrewsbury SB et al., following a double-blind study, advocate for intranasal olanzapine for agitated states requiring antipsychotic treatment.

Intranasal olanzapine reduces adverse effects, patient discomfort, and risks to healthcare professionals.

It is contraindicated in patients at risk of bronchospasm. Early initiation during less severe agitation phases and voluntary administration are highlighted as key factors for success [29].

#### 3.3.5. Inhaled Loxapine

McDowel M et al. defend inhaled loxapine for agitation of psychotic origin, reporting that it shortens the time to crisis control, reduces the need for benzodiazepines and mechanical restraints, and avoids adverse events like QT interval prolongation.

Similarly to olanzapine, inhaled loxapine is contraindicated in patients at risk of bronchospasm. Early administration with patient cooperation is crucial in controlling agitation at its lowest possible level [31].

#### 3.3.6. Other Drugs Suitable for Intranasal Administration

The Emergency Nurses Association (ENA) identifies diazepam, lorazepam, haloperidol, midazolam, and ketamine as suitable for intranasal administration [11].

The Spanish Society of Pediatric Emergency Medicine (SEUP) also supports the intranasal use of diazepam, midazolam, ketamine, and haloperidol [19].

#### 3.3.7. General Recommendations

Tucker C et al. note that, despite the lack of specific formulations for intranasal administration, parenteral formulations achieve good results when used intranasally [33].

The SUMMA procedure manual acknowledges the effectiveness of the intranasal route for managing agitated patients but does not specify which drugs are suitable for this method [5].

See Table 7 for a summary of drugs recommended for intranasal administration in agitated patients.

## 4. Discussion

The present study consolidates existing information on the use of the intranasal route for pharmacological containment in patients experiencing psychomotor agitation—a psychiatric emergency that necessitates immediate and coordinated intervention by a multidisciplinary team to ensure an effective approach [16,28].

Such situations frequently arise in prehospital settings [4], where ensuring the safety of the patient, the environment, and healthcare professionals presents significant challenges [4,8,21]. Rodríguez P.A. underscores the complexity of managing patients with psychomotor agitation, advocating for the early implementation of therapeutic measures at the lowest possible level of agitation to prevent symptom escalation [7]—a perspective endorsed by E. Martínez Larrull et al. [23]. The early recognition of prodromal symptoms is particularly valuable in facilitating prompt therapeutic interventions [17,29,37]. In cases where early intervention is not achieved, the Spanish Consensus of Good Practices advises the adoption of measures to prevent further progression of the condition [38].

A critical aspect of patient management involves identifying whether the agitation has an organic, psychiatric, or mixed origin [16,17,18,20,22,24,25,27,37,38]. The SUMMA procedure manual highlights the importance of distinguishing between agitation, aggressiveness, and violence [5], while P. Sanz Correcher emphasizes the need to differentiate agitation from delirium [22].

Most authors agree on the importance of fostering a therapeutic relationship and creating a safe environment for both the patient and the healthcare team [5,16,17,20,21,23,24,25,34,35,37,38]. Carlos Bibiano Guillén stresses the necessity of maintaining a safe distance, providing a calm environment, removing hazardous objects, and securing exit routes to ensure safety [18]. Similarly, the action protocols of the Government of Aragón [24] and the Medical Emergency Service of La Rioja [21] emphasize the importance of interdisciplinary coordination with state security forces in out-of-hospital contexts.

Once safety measures are in place, treatment should progress through three therapeutic steps: verbal restraint, pharmacological restraint, and physical restraint [5,16,17,21,22,23,26,27,29,30,32,33,35,37,38]. Verbal restraint aims to reduce anxiety [25], reassure the patient [37], and manage the condition [37], as noted in the Riojan Health Service protocol [21] and by J.I. Gállego-Gómez [37]. P. Sanz Correcher recommends offering pharmacological therapy during this phase to alleviate anxiety [22].

For cooperative patients, oral administration is generally preferred, as indicated by E. Martínez Larrull [23], the ANESM and SEEUE societies [35], the Clínica Universitaria de Navarra guide [16], and the Servicio de Emergencias de Andalucía [27]. The choice of administration route—oral, intranasal, or parenteral—should be guided by the patient’s level of cooperation and/or agitation [35]. When verbal de-escalation techniques fail, pharmacological containment becomes essential, as supported by SUMMA [5], the 061 protocol of La Rioja [21], E. Martínez Larrull [23], and the ANESM and SEEUE societies [35], who recommend benzodiazepines, neuroleptics, or their combination.

Several sources, including J.I. Gállego-Gómez et al. [37], the CUN protocols [16], the Government of Aragón [24], the Galician Health Service [25], the Hospital Universitario Príncipe de Asturias [26], SAMUR [17], EPES [27], and the Spanish Consensus of Good Practices [38], concur that combining these drugs achieves faster sedation with fewer side effects [35]. Carlos Bibiano Guillén also supports this approach [18]. However, the Spanish Consensus of Good Practices advocates for monotherapy with short-acting drugs at the lowest effective dose [38].

Mechanical restraint, considered an exceptional measure, is addressed by Alonso Pérez Toribio et al. [34], E. Martínez Larrull et al. [23], Carlos Bibiano Guillén [18], the ANESM and SEEUE societies [35], and the Spanish Society of Psychiatry [38]. The SUMMA procedure manual specifies that mechanical restraint should only be used as a last resort [5], while the 061 protocol of La Rioja advises its application solely in cases of imminent risk to ensure safety [21]. This perspective is shared by J.I. Gállego-Gómez et al. [37] and the CUN action protocol [16]. P. Sanz Correcher highlights the importance of providing appropriate care during the period of mechanical restraint [22], while Carlos Bibiano Guillén advises reevaluating the patient every two hours [18]. The SUMMA manual further stresses the need for vigilance during transport [5].

Regarding intranasal drug administration, the ENA [11], Vargas Velázquez de Castro et al. [32], and the Spanish Society of Pediatric Emergencies [SEUP] [19] identify the atomizer device as the optimal tool, as it produces a fine mist that enhances drug distribution and absorption. Bañuelos-Huerta et al. support this conclusion [36]. However, the ENA [11], Tucker C et al. [33], and the SEUP caution that the presence of mucus, blood, or mucosal alterations can impede absorption. Both the SEUP [19] and SAMUR [17] note that the prior use of vasoconstrictor substances similarly reduces the absorption efficiency. Vargas Velázquez de Castro et al. specifically highlight mucus as a limiting factor [32].

The ENA clinical practice guide emphasizes that the proper execution of the intranasal administration technique is crucial in achieving the desired therapeutic effect [11].

## 5. Conclusions

The research established that the intranasal route is an effective, safe, and straightforward method for administering pharmacological therapy to agitated patients in out-of-hospital settings. It offers nursing professionals a fast and practical alternative to the parenteral route, which is riskier for healthcare providers and more painful for patients, particularly in highly agitated or hostile situations. In cases where cooperation is possible, voluntary oral medication remains the preferred option.

Key takeaways include

1. Advantages of the intranasal route: It is ideal for challenging environments where establishing a therapeutic relationship is difficult.
2. Ease of administration: The technique is simple but requires proper preparation, including the use of an intranasal atomizer for effective drug diffusion and absorption.
3. Medications and guidelines: Drugs like midazolam, haloperidol, ketamine, and olanzapine are suitable for this route. Doses should be divided between the nostrils, with recommended volumes of 0.2–0.3 mL per nostril (up to 1 mL for adults), and repeated every 10–15 min if necessary.

This method effectively addresses the challenges of pharmacological containment in out-of-hospital settings, offering a reliable alternative to parenteral administration.

## 6. Implication for Nursing

The intranasal route offers nurses a fast, safe, and less invasive option for managing agitated patients in out-of-hospital settings, reducing the risks associated with parenteral administration. Proper training on intranasal techniques, including the use of atomizers and dosage guidelines, is essential for effective drug delivery. Familiarity with medications such as midazolam, haloperidol, ketamine, and olanzapine is crucial to safe practice. This method enhances safety in hostile environments while prioritizing patient-centered care. When possible, oral medication remains the preferred option for cooperative patients.

Limitations and bias: A key challenge of the study highlights the opportunity to further explore the availability of specific formulations for intranasal drug administration in Spain. This gap underscores the potential for future research to enhance the practical application of this therapeutic approach.

## Figures and Tables

**Table 1:** Search strategy.

Base	Research Strategy	Results	Selected Article
Pubmed	“Administration, Intranasal/nursing” [Mesh]	2	1
	((drug administration, intranasal [MeSH Terms])) AND (psychomotor agitation [MeSH Terms])	12	2
	(emergency medical services [MeSH Terms])) AND (administration, intranasal [MeSH Terms])	49	1
	((agitation, psychomotor [MeSH Terms]) AND (administration, intranasal [MeSH Terms])) AND (drug therapy [MeSH Terms])	3	0
	(administration, intranasal [MeSH Terms]) AND (alternative method)	95	2
	((agitation, psychomotor [MeSH Terms]) AND (emergency medical services [MeSH Terms]) AND (drug therapy [MeSH Terms]	38	1
Dialnet	Agitated patient	55	3
	Intranasal route	41	1
	Out-of-hospital psychiatric emergencies	4	1
ENFISPO	Agitated patient	14	1
	Intranasal route	5	1
	Out-of-hospital psychiatric emergencies	2	0
	Out-of-hospital emergency medical services	14	0
Google Scholar	Pharmacological treatment and intranasal administration and agitated patient and out-of-hospital medical emergency services	65	2
	“Pharmacological treatment” and “psychomotor agitation”	115	1
	Intranasal atomizer	113	3

**Table 2:** Research question.

Patient	Intervention	Comparison	Outcome
Patients with psychomotor agitation	The use of the intranasal route for the administration of drug therapy	N/A	Improving the quality of care and safety in the out-of-hospital approach

**Table 3:** Steps to follow in the management of a patient presenting with psychomotor agitation.

Analysis of Content																									
Ref.	[5]	[11]	[15]	[16]	[17]	[18]	[19]	[20]	[21]	[22]	[23]	[24]	[25]	[26]	[27]	[28]	[29]	[30]	[31]	[32]	[33]	[34]	[35]	[36]	[37]
Steps to follow	✓		✓	✓	✓		✓	✓	✓		✓	✓	✓	✓			✓				✓	✓		✓	✓
Correct technique		✓		✓		✓													✓	✓					
Possibility of intranasal administration	✓	✓				✓		✓							✓	✓	✓	✓		✓		✓	✓		

**Table 4:** Scope of care.

Scope of Care																	
Ref.	[5]	[16]	[17]	[18]	[20]	[21]	[22]	[23]	[24]	[25]	[26]	[27]	[30]	[34]	[35]	[37]	[38]
Care in prehospital setting	✓		✓		✓	✓			✓	✓		✓	✓				
Care at the hospital level		✓		✓				✓			✓					✓	
Not specified area							✓							✓	✓		✓

**Table 5:** Steps to follow in the approach to an agitated patient (own elaboration).

Ref.	Determining Origin	Safety	Verbal Containment	Pharmacological Containment	Therapeutic Insulation	Mechanical Containment
[6]	✓	✓	✓	✓		✓
[16]	✓	✓	✓	✓		✓
[17]	✓	✓		✓		✓
[18]	✓		✓	✓		✓
[20]	✓	✓	✓	✓		✓
[21]		✓	✓	✓		✓
[22]	✓		✓	✓		✓
[23]		✓	✓	✓		✓
[24]	✓	✓	✓	✓		✓
[25]	✓	✓	✓	✓		✓
[26]			✓	✓		✓
[27]	✓	✓		✓		✓
[30]				✓		
[34]		✓	✓	✓		✓
[35]	✓	✓	✓	✓	✓	✓
[36]	✓	✓	✓	✓		✓
[37]	✓	✓	✓	✓		✓

**Table 6:** Key points for intranasal drug delivery (own elaboration).

Ref.	Maximum Volume	Ideal Volume	Residual Volume	Affects of Absortion				Application Systems			
				Mucus	Blood	Septal Abnormality	Vasoconstrictor Drug	Dripping	Aerosol	Atomiser	Nebulizer
[11]	1 mL	0.2–0.3 mL	---	✓	✓	✓	✓	✓	✓	✓	✓
[17]	1 mL	0.2–0.3 mL	0.1 mL	✓	✓		✓			✓	
[19]		0.2–0.3 mL	0.1 mL	✓	✓	✓	✓			✓	
[32]			0.1 mL							✓	
[33]	1 mL	0.2–0.3 mL	---	✓	✓	✓		✓		✓	

**Table 7:** Drugs indicated for the pharmacological containment of the agitated patient suitable for intranasal administration (own elaboration).

Ref.	Benzodiazepines				Neuroleptics					Others	
	Diazepam	Midazolam	Lorazepam	Dipotassium Chlorazepate	Haloperidol	Chlorpromazine	Levopromazine	Olanzapine	Loxapine	Ketamine	Propofol
[6]											
[11]		✓									
[19]		✓									
[21]		✓									
[28]		✓			✓					✓	
[29]		✓									
[30]		✓									
[31]		✓			✓					✓	
[33]		✓			✓					✓	
[35]		✓									
[36]		✓								✓	

## Data Availability

The raw data will be made available upon contact with the first author. The results are available upon request to the corresponding author.

## Public Involvement Statement

There was no public involvement in any aspect of this research.

## Guidelines and Standards Statement

This manuscript was drafted against the PRISMA guidelines for systematic reviewers.
